# Can cognitive behavioral therapy improve vasomotor symptoms and recurrent depression in postmenopausal women?

**DOI:** 10.1590/1806-9282.20231791

**Published:** 2024-08-16

**Authors:** Leiliane Aparecida Diniz Tamashiro, José Maria Soares-Jr, Joel Renno, José Antônio Orellana Turri, Iara Moreno Linhares, Edmund Chada Baracat, Nilson Roberto de Mello

**Affiliations:** 1 Universidade de São Paulo, Hospital das Clinicas, Institute of Psychiatry, Faculty of Medicine, Women's Mental Health Program - São Paulo (SP), Brazil.; 2 Universidade de São Paulo, Faculdade Medicina, Hospital das Clínicas, Departamento de Obstetrícia e Ginecologia, Disciplina de Ginecologia - São Paulo (SP), Brazil.

**Keywords:** Cognitive behavioral therapy, Menopause, Depression, Recurrent, Cognition

## Abstract

**OBJECTIVE:**

The aim of this study was to evaluate the effectiveness of cognitive behavioral therapy in the treatment of vasomotor, sexual dysfunction, and recurrent depression in postmenopausal women.

**METHODS:**

This prospective, open study evaluated 112 postmenopausal women with vasomotor symptoms. Sexual dysfunction has cultural, social, biological, and emotional issues and divided into two groups: G1, without depression (n=65) and G2, with recurrent depression (n=47). The subjects underwent 12 sessions of in-person cognitive behavioral therapy and 12 sessions of home-based activity over a period of 6 months. They were evaluated at 3 months following the completion of therapy. Depression, memory, and attention-related functions, as well as climactic symptoms, were assessed using a questionnaire.

**RESULTS:**

In the depression questionnaire, the G1 group had a lower initial score than the G2 group (p<0.01). Following 6 months of therapy, both groups had similar improved scores. In the depression questionnaire, the women in group G1 had higher baseline values. In the assessment of vasomotor symptoms, the values in both groups were similar and showed an improvement in vasomotor symptoms after 24 weeks of treatment, but these effects disappeared after the follow-up of 48 weeks in the G2 group. Both groups improved the sexual dysfunction after 24 weeks.

**CONCLUSION:**

Cognitive behavioral therapy may be effective in reducing vasomotor symptoms and ameliorate the sexual dysfunction and recurrent depression in postmenopausal women after 24 weeks of treatment.

## INTRODUCTION

Cognitive behavioral therapy (CBT) in postmenopausal women has been evaluated as a treatment for recurrent depression and sexuality^1,2^. In fact, evidence indicates that CBT has the potential to effectively promote mental and sexual health in clinical trial participants^3,4^. The attention and amnestic function tests are also the most common methods used to evaluate the success of CBT^5^.

Recurrent depression is a disorder characterized by the repeated occurrence of this symptom in the absence of any antecedent independent episodes of mood exaltation and increased energy. This affection also has variable length from a few weeks to a few months, and at least two episodes might have lasted for at least 2 weeks, separated by months^6,7^. This may worsen in the postmenopausal women with vasomotor symptoms^6,7^. In addition, the highest incidence of depression and decline in cognitive functions occurs during this period^5,6^. Difficulties arise in the performance of professional and domicile tasks, which may further negatively reduce emotional stability^6^.

Cognitive behavioral therapy may be an alternative therapy to change behavior and emotional state^7^ as well as has a positive influence on psychic status and vasomotor symptoms^8^. CBT may also affect sexual dysfunction, such as hypoactive sexual desire disorder, sexual arousal dysfunction, orgasmic dysfunction, psychogenic dyspareunia, and sexual dysfunction penetration that correspond to HAOO.2, HA01.03, HA02.02, HA20, and HA40.1 CID 11, respectively^9^. The aim of this study was to evaluate the effectiveness of CBT in the treatment of vasomotor, sexual dysfunction, and recurrent depression in postmenopausal women^10,11^.

## METHODS

We performed a prospective, case-control, open study to assess the changes in vasomotor symptoms and depression in postmenopausal women following CBT. The study was approved by the Institutional Review Board of the Faculty of Medicine of the University of Sao Paulo Research project approved by the Ethics Committee for analysis of CAPPesq Projects and Research at HC-FMUSP under protocol opinion 235.365 (number 235,375), and all subjects provided informed written consent.

Women were evaluated by psychiatrists of the Women's Mental Health Program (ProMulher) Institute of Psychiatry - HCFMUSP without other psychiatric disorders^12,13^. They were evaluated at ProMulher ambulatory second-floor south wing, a private room, for local recruitment psychology, data collection, and CBT sessions, from 1 p.m. to 7 p.m. on Monday and 4 p.m. to 7 p.m. on Thursday. Each session comprised six patients, lasting for 1 h and carried out by a research psychologist. Depressed women in the group with clinical demographic data were verified and recruited from the Climacteric Ambulatory Division of the Discipline of Gynecology IC-HCFMUSP and Women's Mental Health Program (ProMulher) IPq - HCFMUSP.

Inclusion criteria were postmenopausal women between 50 and 60 years of age, not using hormone replacement therapy, being postmenopausal between 2 and 6 years, and having more than 4 years of schooling. Exclusion criteria included primary ovarian insufficiency, hysterectomy with bilateral annexectomy, use of psychotropic drugs in the last 6 months prior to evaluation, impairment in cognitive processes, other psychiatric disorders, low intelligence quotient (IQ) (<79), severe and moderate depression with psychotropic treatment, and uncompensated endocrinopathies.

### Groups

A total of 269 women were screened, of which 82 (55.0%) did not meet the inclusion criteria, 45 (30.2%) were evaluated (fear of cognitive degeneration), 13 (8.72%) were excluded (less than 75% attendance), and 9 (6.0%) left without justification. Women completed the program (n=120), but when called for re-evaluation, 8 (6.6%) changed their residence (city and state). A total of 112 women in the postmenopausal study were divided into two groups: G1 without depression (n=65) and G2 with recurrent depression (n=47).

### Procedure

Participants gave written informed consent. The interviews were conducted face-to-face and recorded for transcription. The mean length of all evaluation through the questionnaires was 90 min±10 min.

### Interview guide

At the initial visit, women's clinical history was ascertained to establish baseline clinical data. The evaluation of depression/anxiety and climacteric symptoms was performed using the Beck Depression Inventory (BDI)^14^ and Greene Climacteric Scale^15^, respectively. A diagnostic questionnaire to characterize recurrent depression was applied. The patients underwent neuropsychological analysis to evaluate attention and memory before completing the specific neuropsychological questionnaires.

A questionnaire structured with demographic factors (age, marital status, education, professional occupation) was used to assess clinical pretreatment status. It also included menstrual, gynecological, obstetric, puerperal, psychiatric, and sexual history, as well as information about specific complaints during their premenstrual period and following menopause.

The BDI^14^ questionnaire has 21 groups of affirmations with four items and evaluates the presence and intensity of depressive symptoms. It also describes how the individual has felt in the previous week, including the day of the evaluation, using a cutoff of 18 to indicate moderate depression.

The validated GCS^15^ comprises psychological symptoms of anxiety and depression, physical (somatic) and vasomotor symptoms, and sexual dysfunction in four degrees of intensity: symptoms absent, mild—not bothersome without interfering with everyday life, and intense—bothersome and interfere with daily life. It also contains 6 questions for anxious symptoms, 5 for depressive symptoms, 7 for somatic symptoms, 2 for vasomotor symptoms, and 1 for sexual symptoms, with a total of 21 questions (1. heart beating quickly or strongly; 2. feeling tense or nervous; 3. difficulty in sleeping; 4. excitable; 5. attacks of anxiety, and panic; 6. difficulty in concentrating; 7. feeling tired or lacking in energy; 8. loss of interest in most things; 9. feeling unhappy or depressed; 10. crying spells; 11. irritability; 12. feeling dizzy or faint; 13. pressure or tightness in head; 14. parts of body feel numb; 15. headaches; 16. muscle and joint pains; 17. loss of feeling in hands or feet; 18. breathing difficulties; 19. hot flushes; 20. sweating at night; and 21. loss of interest in sex). The following scores are based on the symptoms: 0 (absence); 1 (mild); 2 (moderate), and 3 (intense). The total maximum score is 63 points for all questions. The five domains of GCS are anxiety (#1-#5 questions), depression (#6-#11 questions), somatic symptoms (#12-#18 questions), vasomotor symptoms (#19 and #20), and sexual function (#21).

The questionnaire on Women's Mental Health (QSM)^16^ depicts the quality of life, assessing physical and mental symptoms, specifically in women of middle age, period of life between maturity and old age. It is grouped into nine domains that evaluate somatic symptoms, depressive mood, concentration/memory problems, anxiety/fear, sexual satisfaction, vasomotor symptoms, sleep disorders, menstrual changes, and attractiveness.

The 20-item Psychiatric Morbidity Scale (SRQ 20)^17^ evaluates the presence of nonpsychotic psychiatric disorders in the population in several countries of different cultures using a cutoff of 8 for women.

The Wechsler Abbreviated Scale Intelligence^18^ was used to estimate IQ. The matrices contain 35 groups of figures with an incomplete grid model to be examined and completed with one correct choice out of five possibilities. Vocabulary contains 42 items representing verbal knowledge. The score was transformed into a weighted note, with its summation providing the total score and the estimated IQ.

### Neuropsychological

The Stroop Color Word Test (SCWT)^19^ evaluates selective attention, inhibitory control, and executive functions, and is composed of three cards with different levels of difficulty. The colors are distributed in six series in a random manner. Evaluation is by execution time, punctuated by mean and standard deviation, including errors.

The Trail Making Test (TMT)^20^ evaluates the speed of attention, sequencing, mental flexibility, visual tracking, and motor function. Part A involves the collection of randomly distributed numbers 1-25. Part B involves an alternate sequence of numbers and letters, numbers 1-13 and letters A-M, distributed in a random order. The evaluation is by execution time, punctuated by means and standard deviation, including errors.

The Numbers and Letters (sub-scale)—Wechsler Adult Intelligence Scale-III^21^ subtest evaluates working memory. It is composed of a list of numbers and letters disordered with seven items and three sequences each. After reading each item, you must sort them alphabetically first and then numerically without making mistakes.

The Digits—Direct and Reverse—Digit Span in Wechsler Memory Scale-Revised^22^ test is divided into two parts. In the first part, the direct order is related to attention, with seven series of numbers, with two attempts each. In the second part, the inverse order is linked to working memory, and executive functions consist of six series of numbers, with two attempts each. The gross score is obtained and consequently the percentile^23^.

The Digital Windows—Visuals—Visual Subtests—Wide Range Assessment of Memory and Learning. Second Edition—WRAML II^24^ evaluates immediate memory and visuospatial learning. A plate is used with nine patient-cast circles, randomly numbered from 1 to 9 for the applicator. Following the same sequence carried out by the applicator, corrections and errors are noted on the answer sheet.

The List of Words—Verbal Subtest—Wide Range Assessment of Memory and Learning—WRAML II^24^ evaluates short-term verbal memory and systematic verbal learning ability. There is a list of 16 words to be repeated. The task repeats itself four times and retrieves them later, evaluating the retention in its memory.

### Cognitive behavioral therapy procedure

The CBT procedure was done the next day of questionnaire application with a group of six women following the protocol described in other study^1^. The CBT for each group was performed each 15 days during 24 weeks. Specific CBT exercises help empty stored negative feelings and emotions^4,11^. Through test of amnestic functions and attention, it allows us to indicate a better treatment strategy. The focus of CBT is to change one's behavior and reinforce the emotion^1^. The length of each CBT was 1 year. Two facilitators participated in each CBT. The CBT was held in the specific room only for this procedure in the Women's Mental Health Program (ProMulher) IPq - HCFMUSP.

Breathing exercises were performed before each CBT session and at home. Each woman was evaluated for her breathing pattern. This exercise brought calm, balance, better concentration, reducing anxiety^25–27^. Relaxation exercises bring a better quality of sleep and a decrease in daily stress^1,11^.

### Evaluation

The evaluations of effectiveness were performed at (a) baseline (before CBT), (b) after 24 weeks of CBT (at the end of procedure), and (c) 24 weeks after the end of procedure (follow-up—48 months after baseline).

### Statistical analysis

The sample size and power estimation were calculated based on the mean and standard deviation differences between the WHQ scores in the pre- and post-treatment group in women with depression and menopausal symptoms (published) in the literature^28,29^. With a power of 90% and a significant p-value of 5%, the minimum number of patients calculated for this study was 89. With the inclusion of 120 initial and final 112 patients, the power of this study was 97 and 95%, respectively.

Student's t-test or the Mann-Whitney test was used, according to the data distribution. In the proportion of variables, Fisher's exact test or chi-square test was used. Repeated-measures ANOVA or Kruskal-Wallis was used for comparison among baseline, 24 months, and follow-up in the same group. The tests were performed with a significance level of 5%. Spearman´s correlation test was applied for evaluating the influences of amnestic and attention tests on the Green domains, BDI, and SRQ-20 scale.

## RESULTS

Table 1 summarizes the clinical demographic data of the two groups of participants. There were also no significant differences between the two groups in the clinical demographic data of patients.

**Table 1:** Clinical demographic data of patients.

		G1: no depression (n=65)	G2: depression (n=47)	p
Age (M /SD)		55.5 (2.7)	54.8 (3.1)	0.72
Education (n)				
	Middle school	05 (7.7%)	03 (6.4%)	
	High school	17 (26.2%)	21 (44.7%)	
	University degree	43 (66.2%)	23 (48.9%)	0.88
Marital status (n)				
	Single	13 (20%)	10 (21.3%)	
	Married	26 (40%)	27 (57.4%)	
	Divorced	22 (33.8%)	06 (12.8%)	
	Widowed	04 (6.2%)	04 (8.5%)	0.55
Professional status (n)				
	Work	33 (50.8%)	33 (70.1%)	
	Pensioned off	14 (21.5%)	10 (21.3%)	
	Housewife	18 (27.7%)	04 (8.5%)	0.66
Body mass index (kg/m^2^)				
	< 25	10 (15.4%)	07 (14.9%)	
	≥25	55 (84.6%)	40 (85.1%)	0.94
Physical exercise (n)				
	Yes	14 (21.5%)	09 (19.1%)	
	No	51 (78.5%)	38 (80.9%)	0.75

n: number of patients; G: group; M: mean; SD: standard deviation; p: p-value.

Table 2 summarizes the evaluation of amnestic and attention tests in both groups. In the SCWT-"D", SCWT-Erros-D", SCWT-W, SCWT-Erros-W, SCWT-CV, and SCWT-Erros-CW, there was a significant increase in values at 24 weeks in G2 comparing baseline and after 24 weeks (p<0.05). The G1 presented improved in SCWT-W and SCWT-CW (p<0.05). There is no difference in the comparison of baseline and 24 weeks of CBT when comparing both groups. After the follow-up evaluation (48 weeks), the values of G2 were higher than the ones of G1 (p<0.05).

**Table 2:** Evaluation of amnestic and attention tests in both groups.

Evaluation/time weeks	G1: no depression (n=65)			G2: recurrent depression (n=47)			Comparison among periods in G1			Comparison among periods in G2			Comparison between groups in different periods		
	Basal	24	48	Basal	24	48	Basal X 24	24×48	Basal X 48	Basal X 24	24×48	Basal X 48	Basal	24	48
SCWT-"D"¹	17.48 (3.86)	18.72 (3.72)	32.40 (7.89)	17.40 (3.61)	20.70 (5.11)	35.80 (9.41)	NS	<0.001	<0.001	NS	<0.001	<0.001	NS	NS	<0.05
SCWT-Erros-"D"¹	0.27 (0.64)	0.24 (0.55)	1.64 (1.84)	0.17 (0.43)	0.27 (0.57)	2.14 (2.43)	NS	<0.001	<0.001	NS	<0.001	<0.001	NS	NS	NS
SCWT -"W"¹	14.47 (2.85)	17.72 (3.82)	29.91 (7.80)	17.40 (3.61)	20.70 (5.11)	35.80 (9.41)	<0.05	<0.001	<0.001	NS	<0.001	<0.001	NS	NS	<0.001
SCWT-Erros- "W"¹	0.07 (0.26)	0.01 (0.12)	0.10 (1.60)	0.02 (0.14)	0.06 (0. 24)	0.85 (1.23)	NS	<0.001	<0.001	NS	<0.001	<0.001	NS	NS	NS
SCWT-"CW"¹	13.48 (2.67)	16.73 (3.10)	29.02 (7.7)	13.69 (2.14)	17.98 (2.58)	30.27 (6.79)	<0.01	<0.001	<0.001	<0.005	<0.001	<0.001	NS	NS	NS
SCWT-Erros- "CW"¹	0.01 (0.12)	0.10 (0.31)	0.53 (1.22)	0.0	0.08 (0.28)	0.48 (1.1)	NS	<0.01	<0.01	NS	NS	<0.05	NS	NS	NS
TMT -"A"¹	1.10 (14.28)	24.46 (8.00)	27.54 (7.12)	31.87 (19.88)	25.05 (15.67)	28.67 (8.28)	NS	NS	NS	<0.05	<0.01	NS	NS	NS	NS
TMT- Erros- "A"¹	0.23 (0.63)	0.10 (0.35)	0.13 (0.42)	0.02 (0.14)	0.04 (0.20)	0.14 (0.41)	NS	NS	NS	NS	NS	NS	NS	NS	NS
TMT -"B"¹	76.84 (23.09)	53.39 (14.02)	63.58 (16.39)	84.14 (36.81)	54.17 (17.24)	61.93 (14.65)	<0.001	NS	<0.01	<0.001	NS	<0.001	NS	NS	NS
TMT- Erros-"B"¹	1.16 (1.45)	0.86 (1.04)	0.46 (0.88)	0.68 (0.95)	0.53 (0.77)	0.40 (0.82)	NS	NS	<0.05	NS	NS	NS	NS	NS	NS
Number and letters^1^	8.24 (2.53)	11.02 (2.25)	14.61 (2.00)	7.34 (2.28)	11.17 (2.11)	13.91 (2.26)	<0.001	<0.001	<0.001	<0.001	<0.001	<0.001	NS	NS	NS
Span digts - direct^2^	10 [4-12]	11 [5-12]	11 [7-12]	10 [4-12]	11 [7-12]	12 [6-12]	NS	NS	<0.001	<0.001	NS	<0.001	NS	NS	NS
Indirect digits^2^	5 [2-12]	7 [2-12]	8 [3-12]	5 [2-11]	8 [2-12]	9 [3-12]	<0.01	NS	<0.001	<0.001	NS	<0.001	NS	NS	NS
Digital windows^1^	6.23 (2.47)	9.10 (2.97)	7.86 (2.42)	6.14 (2.53)	9.10 (2.01)	7.68 (2.26)	<0.001	NS	<0.01	<0.001	NS	0.05	NS	NS	NS
List of words^2^	8 [3-14]	10 [7-16]	11 [6-17]	8 [3-14]	9 [6-15]	12 [9-19]	<0.001	NS	<0.001	NS	<0.001	<0.001	NS	NS	NS
List of words recovery^2^	8 [0-15]	11 [4-16]	14 [5-16]	7 [0-15]	10 [5-16]	14 [7-16]	<0.001	<0.05	<0.001	<0.05	<0.001	<0.001	NS	NS	NS

SCWT: Stroop Color Test; TMT: Trail Making Test.

1 Mean (standard deviation) and unpaired Student's t-test was used. Repeated-measures ANOVA and post-hoc Bonferroni tests were applied for comparison in the same group among periods.

2 Median [interquartile range] and Mann-Whitey test were used. Repeated-measures Kruskal-Wallis and post-hoc Dunn tests were applied for compassion in the same group.

The attention test results are summarized in Table 2. The values of TMT-B, number and letters, indirect digits, digital windows, list of words, list of words recovery of G1 increased after 24 weeks of CBT and maintained after follow-up of 48 weeks (24 weeks without CBT). The G2 group presented an improved TMT-A and other parameters, except those of TMT-Erros A, TMT-Erros B, and list of words. There were no differences in comparison between both groups in relation to baseline, 24 weeks of treatment, and 48 weeks of follow-up (24 weeks without CBT).

The BDI, SRQ-20, and Domains of Scale Greene are summarized in Table 3. The BDI baseline values of G2 (recurrent depression) were higher than those of G1 (no depression). The baseline values were 12.14 (3.93) and 25.21 (4.62) for G1 and G2, respectively (p<0.001). The scores of BDI decreased in both groups after 24 weeks of treatment and follow-up. The comparison between the groups did not find any differences after 24 weeks of treatment or during the follow-up period. Similar results were observed using the SRQ-20 scale. In relation to domains of Scale Greene, the anxiety, depression, sexual dysfunction, and physical symptoms at baseline in G2 were significantly superior to those of G1 (p<0.05), except the vasomotor symptom domain. Both groups presented significant improvements in all domains after 24 weeks of treatment, except the physical domain, where only the G2 group presented a significant decrease in this parameter (p<0.01). The anxiety and depression domain values of G2 were lower than those of G1 (p<0.01). The values of depression and vasomotor symptom domain of G1 during follow-up were significantly lower than baseline. The values of all domains of G2 during the follow-up were significantly different compared with baseline, expect the vasomotor symptom domain. No differences in all domains were found between the groups during the follow-up.

**Table 3:** Results of Beck Depression Inventory, SRQ-20, and domains of the Greene Climacteric Scale.

G1: no depression (n=65)					G2: recurrent depression (n=47)			Comparison among periods in G1			Comparison among periods in G2			Comparison between groups in different periods		
Questionnaires or domains		Basal	24 weeks	48 weeks	Basal	24 weeks	48 weeks	Basal X 24 weeks	24 weeks X 48 weeks	Basal x 48 weeks	Basal X 24 weeks	24 weeks X 48 weeks	Basal x 48 weeks	Basal	24 weeks	48 weeks
BDI^1^		12.14 (3.93)	5.26 (2.78)	8.10 (4.37)	25.21 (4.62)	6.10 (2.83)	8.04 (3.94)	<0.001	<0.05	<0.001	<0.001	NS	<0.001	<0.001	NS	NS
SRQ-20¹		7.78 (4.40)	4.49 (3.84)	3.89 (2.84)	12.55 (3.84)	6.10 (4.39)	4.00 (2.96)	<0.001	NS	<0.001	<0.001	NS	<0.001	<0.001	NS	NS
Domains of Greene Climacteric Scale																
	Anxiety¹	7.12 (3.47)	3.70 (2.44)	4.92 (3.27)	9.55 (2.94)	5.61 (3.19)	5.63 (2.90)	<0.01	NS	NS	<0.01	NS	<0.01	<0.01	<0.05	NS
	Depression^1^	8.52 (4.78)	2.47 (1.89)	3.38 (3.08)	12.94 (3.71)	5.63 (4.58)	3.83 (2.83)	<0.01	NS	<0.01	<0.01	NS	<0.01	<0.01	<0.01	NS
	Sexual dysfunction^1^	1.84 ±1.12	2.30 ±0.98	1.60 ±1.04	2.36 ±1.03	1.16 ±0.83	1.59 ±0.99	<0.01	<0.01	NS	<0.01	NS	<0.01	<0.05	NS	NS
	Vasomotor symptoms^1^	2.80 ±2.15	1.10 ±1.58	0.67 ±1.07	1.66 ±2.13	1.06 ±1.35	0.72 ±1.05	<0,01	NS	<0,01	<0.01	NS	NS	NS	NS	NS
	Physical symptoms^1^	5.76 ±4.24	4.04 ±3.17	4.44 ±3.56	8.93 ±5.01	3.23 ±2.75	4.12 ±2.93	NS	NS	NS	<0.01	NS	<0.01	<0.01	NS	NS
	Total^1^	25.85 ±11.43	13.55 ±7.71	14.95 ±8.46	36.60 ±11.21	18.00 ±9.51	15.96 ±7.24	<0.01	NS	<0.01	<0.01	NS	<0.01	<0.01	NS	NS

BDI: Beck's Depression Inventory; SRQ: Self-Reporting Questionnaire;

1 Mean (standard deviation) and unpaired Student's t-test were used. Repeated-measures ANOVA and post-hoc Bonferroni tests were applied for comparison in the same group among periods.

### Correlation test

We assessed whether the SCWT-D and SCWT-W scales would be influenced by other conditions such as the Greene Climacteric Scale and SRQ-20. At baseline, the SCWT-W scale (Spearman's rho=0.3939, p<0.01), the total Greene Climacteric Scale (Spearman's rho=0.2776, p<0.01), and the SRQ-20 scale (Spearman's rho=0.3200, p<0.01) were all positively correlated with physical symptoms. At 24 weeks, the SCWT-D variable was correlated with vasomotor symptoms (Spearman's rho=0.2579, p<0.01). The SRQ-20 scale correlated with vasomotor symptoms (Spearman's rho=0.194, p<0.01), while the SCWT-W variable was only marginally related to vasomotor symptoms (Spearman's rho=0.1887, p<0.01). After 48 weeks, the SCWT-D variable score was related to vasomotor symptoms (Spearman's rho=0.2571, p<0.01). The SCWT-W variable was similarly correlated to vasomotor symptoms.

## DISCUSSION

Recurrent depression is a major cause of professional incapacitation and disruption of family ties, leading to global affective and professional problems^10^. In addition, when there are other associated stressors, such as postmenopausal vasomotor waves, the recurrence and intensity of this condition can increase greatly^8^. Our present findings show that CBT can reduce the symptoms associated with recurrent depression as well as the occurrence of menopausal symptoms such as hot flashes and sexual dysfunction. The amnestic parameters have a positive correlation with the improvement in recurrent depression.

Hot flashes interfere with sleep, quality of life, and recurrence and severity of depressive symptoms^27^, which encompass not only mood changes but also a deficiency in concentration and difficulty in carrying out daily activities^26^. In our study with CBT, the most patients’ frequent dysfunctional automatic thoughts were (a) assuming responsibility for some external situation when in fact others were actually responsible, (b) interpreting their experiences as being totally good or totally bad, (c) concluding that she knows what others are thinking of her and focuses on only one aspect of the situation that she is facing while other aspects are ignored, (d) rejecting positive information about themselves or a situation, and only seeing the negative, (e) maximizing negative experiences while positive ones are minimized, (f) placing a global and rigid label on someone or a situation instead of evaluating it as a whole, (g) interpreting events in terms of how they should be instead of focusing on how they are, and (h) believing that what has happened may recur and will be terrible, intolerable, or unbearable^27^. All these items have negative consequences after CBT.

Our study had some limitations: (a) no control group with recurrent depression without CBT; (b) subjects also had an unsatisfactory response to convectional drug therapy and, therefore, may be a select group; (c) we remain unaware of the duration of gains achieved by CBT; (d) long period of follow-up to check whether there is the residual benefit of CBT; and (e) vasomotor and sexual dysfunction were evaluated using the Greene Climacteric Scale. Therefore, further study using specific questionnaire on sexuality and digital vasomotor symptom registration is necessary to evaluate the real effects of CBT.

The strengths of our study are the evaluation of amnestic and attention parameters on the results of CBT and the follow-up of patients: the benefits on anxiety, depression, and physical symptoms of recurrent depression may remain after CBT therapy.

## CONCLUSION

Cognitive behavioral therapy may be effective in reducing vasomotor symptoms and ameliorating the sexual dysfunction and recurrent depression in postmenopausal women after 24 weeks of treatment.
